# Skeletal Muscle Fiber Type: Influence on Contractile and Metabolic Properties

**DOI:** 10.1371/journal.pbio.0020348

**Published:** 2004-10-12

**Authors:** Juleen R Zierath, John A Hawley

## Abstract

Zierath and Hawley discuss how different fiber types affect muscle metabolism and what the signals are that regulate muscle phenotype

Skeletal muscle demonstrates a remarkable plasticity, adapting to a variety of external stimuli (Booth and Thomason 1991; Chibalin et al. 2000; Hawley 2002; Flück and Hoppeler 2003), including habitual level of contractile activity (e.g., endurance exercise training), loading state (e.g., resistance exercise training), substrate availability (e.g., macronutrient supply), and the prevailing environmental conditions (e.g., thermal stress). This phenomenon of plasticity is common to all vertebrates (Schiaffino and Reggiani 1996). However, there exists a large variation in the magnitude of adaptability among species, and between individuals within a species. Such variability partly explains the marked differences in aspects of physical performance, such as endurance or strength, between individuals, as well as the relationship of skeletal muscle fiber type composition to certain chronic disease states, including obesity and insulin resistance.

In most mammals, skeletal muscle comprises about 55% of individual body mass and plays vital roles in locomotion, heat production during periods of cold stress, and overall metabolism (Figure 1). Thus, knowledge of the molecular and cellular events that regulate skeletal muscle plasticity can define the potential for adaptation in performance and metabolism, as well as lead to the discovery of novel genes and pathways in common clinical disease states.

**Figure 1:**
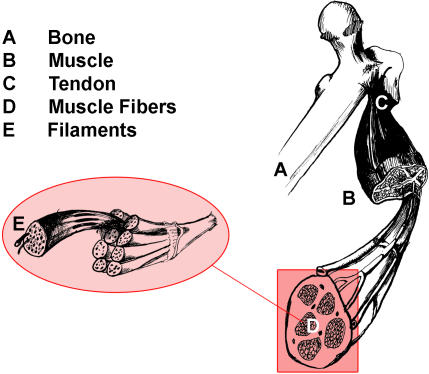
Anatomy of a Skeletal Muscle Individual bundles of muscle fibers are called fascicles. The cell membrane surrounding the muscle cell is the sarcolemma, and beneath the sarcolemma lies the sarcoplasm, which contains the cellular proteins, organelles, and myofibrils. The myofibrils are composed of two major types of protein filaments: the thinner actin filament, and the thicker myosin filament. The arrangement of these two protein filaments gives skeletal muscle its striated appearance.

## How Is Skeletal Muscle Fiber Type Classified?

Much of our early understanding of the plasticity of skeletal muscle has been derived from studies undertaken by exercise physiologists (e.g., Holloszy 1967). With the application of surgical techniques to exercise physiology in the late 1960s (Bergstrom and Hultman 1966), it became possible to obtain biopsy samples (∼150 mg) of human skeletal muscle, and by means of histological and biochemical analyses, specific morphological, contractile, and metabolic properties were identified. In 1873, the French anatomist Louis Antoine Ranvier had already observed that some muscles of the rabbit were redder in color, and contracted in a slower, more sustained manner, than paler muscles of the same animal. These early observations formed the basis of the classical terminology of red and white muscle fibers, which was subsequently found to be related to myoglobin (an iron-containing oxygen-transport protein in the red cells of the blood) content (Needham 1926). Based upon histochemical staining (Engel 1962), muscle fibers are now commonly distinguished as slow-twitch (ST), which stain dark or red, and fast-twitch (FT), which stain light or pale. In humans, a further subdivision of the FT fibers is made (Brooke and Kasier 1970), whereby the more aerobic (or oxidative) FT fiber is designated FT_a_, and the more anaerobic (glycolytic) fiber is termed FT_b_. Under aerobic conditions (sufficient oxygen supply to the working muscles), energy is produced without the production of lactate. Under anaerobic conditions (insufficient oxygen supply to the working muscles), energy is produced via the glycolytic pathway, which results in lactate accumulation and in turn limits anaerobic exercise. Thus, muscle fibers can be classified in terms of contractile and metabolic properties (Table 1).

**Table 1:**
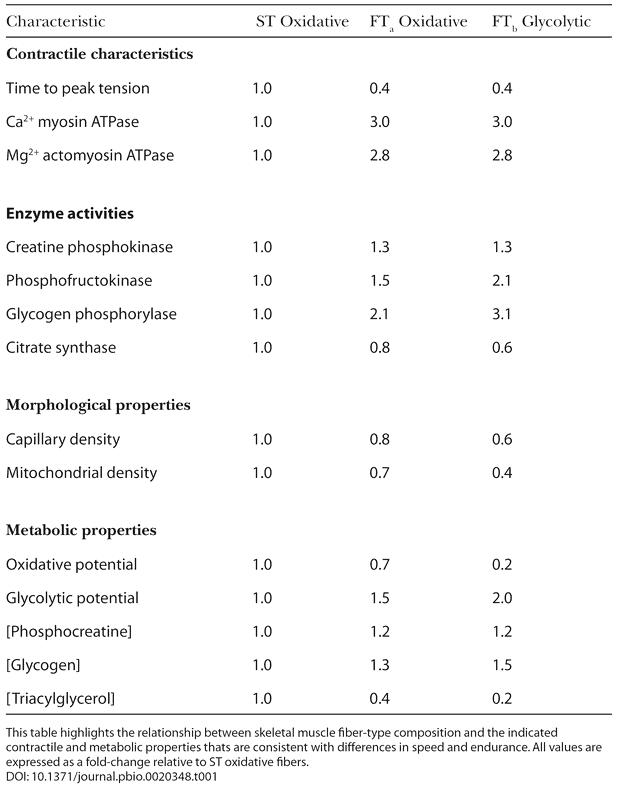
Contractile Characteristics, Selected Enzyme Activities, and Morphological and Metabolic Properties of Human Skeletal Muscle Fiber Types

This table highlights the relationship between skeletal muscle fiber-type composition and the indicated contractile and metabolic properties thats are consistent with differences in speed and endurance. All values are expressed as a fold-change relative to ST oxidative fibers

All individuals have different capacities to perform aerobic or anaerobic exercise, partly depending on their muscle fiber composition. In untrained individuals, the proportion of ST fibers in the *vastus lateralis* muscle (the largest of the quadriceps muscles and the most commonly studied muscle in humans), is typically around 55%, with FT_a_ fibers being twice as common as FT_b_ fibers (Saltin et al. 1977). While marked differences in the metabolic potentials between FT_a_ and FT_b_ fibers are observed in untrained humans, the absolute level for the activities of oxidative and glycolytic enzymes in all fiber types is large enough to accommodate substantial aerobic and anaerobic metabolism (Saltin et al. 1977). While there is a large degree of homogeneity within individual skeletal muscles from rodents (Delp and Duan 1996), this is not the case for humans (Saltin et al. 1977). The dramatic heterogeneity of fiber type composition between people may explain their remarkable variation in exercise performance.

## Does Muscle Fiber Type Composition Influence Athletic Performance?

During the 1970s and 1980s, it was popular to determine the muscle fiber composition of athletes from different sports events. These studies revealed that successful endurance athletes have relatively more ST than FT fibers in the trained musculature (Costill et al. 1976; Fink et al. 1977; Saltin et al. 1977). In contrast, sprinters have muscles that are composed predominantly of FT fibers (Costill et al. 1976). Accordingly, the belief that muscle fiber type can predict athletic success gained credibility. In particular, the notion that the proportion of ST fibers might be a factor governing success in endurance events was proposed (Gollnick et al. 1972; Costill et al. 1976).

**Figure 2:**
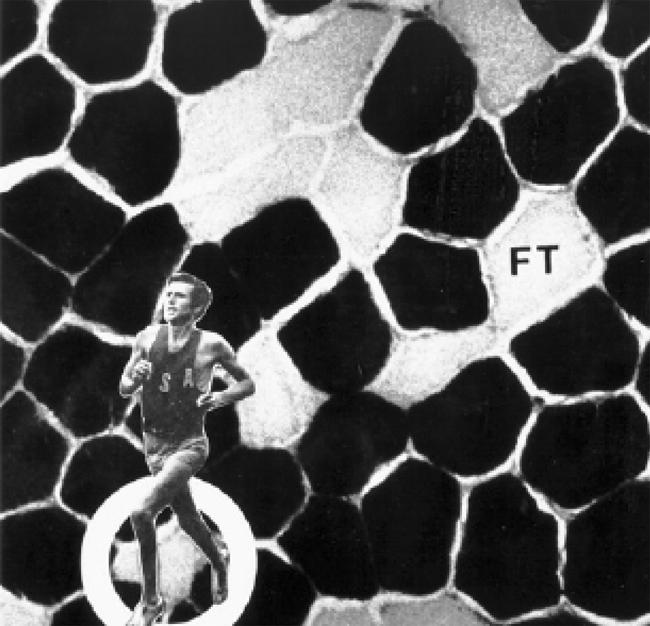
Microscopic View of the *Gastrocnemius* Skeletal Muscle from a World-Class Marathon Runner, Frank Shorter (Olympic Gold Medalist, 1972; Olympic Silver Medalist, 1976) The darkly stained fibers are relatively slow in contractile rate and are ST. These fibers demonstrate a higher aerobic (oxidative) capacity and a lower anaerobic (glycolytic) potential than the lighter stained FT fibers. Shorter's muscle contains approximately 80% ST fibers. Reproduced with kind permission from David L. Costill and William J. Fink.

In this regard, the results of Fink et al. (1977) are important. These researchers determined the fiber composition from the *gastrocnemius* muscle (the muscle of the calf of the leg) of 14 elite male long distance runners, 18 good (but not world-class) male long distance runners, and 19 untrained men. The elite group included Olympic medal winners (Figure 2) and American record holders at the time. Muscle from the elite runners contained a larger proportion of ST fibers than either the good runners or the untrained men (79.0% ± 3.5% versus 61.8% ± 2.9% versus 57.7% ± 2.5% respectively; *p* < 0.05). The values found for several of the elite runners were the highest observed in human muscle (> 92% ST). Moreover, the ST fibers from the elite runners were 29% larger than FT fibers (*p* < 0.05), and both ST and FT fibers were larger in the good runners than in the untrained men. Because of the marked hypertrophy (bulk increase) of the ST fibers in the elite runners, the cross-sectional area composed of these fibers was greater than either the good runners or the untrained subjects (82.9% ± 3.1% versus 62.1% ± 2.6% versus 60.0% ± 2.7% respectively; *p* < 0.05). When the data from the elite and good runners was combined, a positive correlation between the proportion of ST fibers and the best 6-mile performance time was noted (*r* = −0.62, *p* < 0.05).

However, fiber type alone did not determine the performances of the elite athletes. For example, two athletes with similar best times for the 42.2 km marathon distance (approximately 2 hr 18 min) had 50% versus 98% ST muscle fibers. Subsequent work (Foster et al. 1978) revealed that endurance running performance was better related to an athlete's maximal O_2_ uptake (VO_2max_; *r*= −0.84, −0.87, and −0.88 for 1-, 2-, and 6-mile times, respectively). Indeed, while an athlete's muscle fiber type is an important morphological component and is related to several contractile and metabolic properties (see Table 1), other physiological factors (e.g., VO_2max_, maximal cardiac output, and speed/power output at the lactate threshold) are more likely to determine the upper limits of endurance capacity (Coyle 1995; Hawley and Stepto 2001).

## Do Alterations in Skeletal Muscle Fiber Type Contribute to Metabolic Disease?

The close coupling between muscle fiber type and associated morphological, metabolic, and functional properties is not confined to athletic ability. Insulin sensitivity also correlates with the proportion of ST oxidative fibers (Lillioja et al. 1987). Specifically, insulin-stimulated glucose transport is greater in skeletal muscle enriched with ST muscle fibers (Henriksen et al. 1990; Song et al. 1999; Daugaard et al. 2000), thus priming ST muscle for accelerated glucose uptake and metabolism. A shift in fiber distribution from ST to FT fibers gives rise to altered activities of key oxidative and glycolytic enzymes (Pette and Hofer 1980). Indeed, the ratio between glycolytic and oxidative enzyme activities in the skeletal muscle of non-insulin-dependent diabetic or obese individuals is related to insulin resistance (Simoneau et al. 1995; Simoneau and Kelley 1997). Similarly, with ageing and physical inactivity, two other conditions associated with ST-toFT fiber-type transformation, oxidative capacity and insulin sensitivity, are diminished (Papa 1996).

## Genes That Define Skeletal Muscle Phenotype

**Figure 3:**
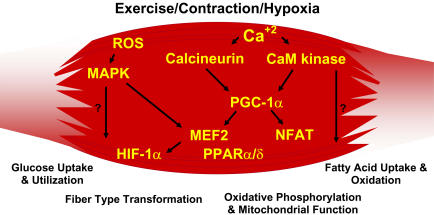
Exercise-Included Signaling Pathways in Skeletal Muscle That Determine Specialized Characteristics of ST and FT Muscle Fibers Contraction-induced changes in intracellular calcium or reactive oxygen species provide signals to diverse pathways that include the MAPKs, calcineurin and calcium/calmodulin-dependent protein kinase IV to activate transcription factors that regulate gene expression and enzyme activity in skeletal muscle.

Skeletal muscle fiber-type phenotype is regulated by several independent signaling pathways (Figure 3). These include pathways involved with the Ras/mitogen-activated protein kinase (MAPK) (Murgia et al. 2000), calcineurin (Chin et al. 1998; Naya et al. 2000), calcium/calmodulin-dependent protein kinase IV (Wu et al. 2002), and the peroxisome proliferator γ coactivator 1 (PGC-1) (Lin et al. 2002). The Ras/MAPK signaling pathway links the motor neurons and signaling systems, coupling excitation and transcription regulation to promote the nerve-dependent induction of the slow program in regenerating muscle (Murgia et al. 2000). Calcineurin, a Ca^2+^/calmodulin-activated phosphatase implicated in nerve activity-dependent fiber-type specification in skeletal muscle, directly controls the phosphorylation state of the transcription factor NFAT, allowing for its translocation to the nucleus and leading to the activation of slow-type muscle proteins in cooperation with myocyte enhancer factor 2 (MEF2) proteins and other regulatory proteins (Chin et al. 1998; Serrano et al. 2001). Calcium-dependent Ca^2+^/calmodulin kinase activity is also upregulated by slow motor neuron activity, possibly because it amplifies the slow-type calcineurin-generated responses by promoting MEF2 transactivator functions and enhancing oxidative capacity through stimulation of mitochondrial biogenesis (Wu et al. 2002).

PGC1-α, a transcriptional coactivator of nuclear receptors important to the regulation of a number of mitochondrial genes involved in oxidative metabolism, directly interacts with MEF2 to synergistically activate selective ST muscle genes and also serves as a target for calcineurin signaling (Lin et al. 2002; Wu et al. 2001). New data presented in this issue of *PLoS Biology* (Wang et al. 2004) reveals that a peroxisome proliferator-activated receptor δ (PPARδ)-mediated transcriptional pathway is involved in the regulation of the skeletal musclefiber phenotype. Mice that harbor an activated form of PPARd display an “endurance” phenotype, with a coordinated increase in oxidative enzymes and mitochondrial biogenesis and an increased proportion of ST fibers. Thus—through functional genomics—calcineurin, calmodulin-dependent kinase, PGC-1α, and activated PPARδ form the basis of a signaling network that controls skeletal muscle fiber-type transformation and metabolic profiles that protect against insulin resistance and obesity.

The transition from aerobic to anaerobic metabolism during intense work requires that several systems are rapidly activated to ensure a constant supply of ATP for the working muscles. These include a switch from fat-based to carbohydrate-based fuels, a redistribution of blood flow from nonworking to exercising muscles, and the removal of several of the byproducts of anaerobic metabolism, such as carbon dioxide and lactic acid. Some of these responses are governed by transcriptional control of the FT glycolytic phenotype. For example, skeletal muscle reprogramming from a ST glycolytic phenotype to a FT glycolytic phenotype involves the Six1/Eya1 complex, composed of members of the Six protein family (Grifone et al. 2004). Moreover, the Hypoxia Inducible Factor-1α (HIF-1α) has been identified as a master regulator for the expression of genes involved in essential hypoxic responses that maintain ATP levels in cells. In this issue of *PLoS Biology* (Mason et al. 2004), a key role for HIF-1α in mediating exercise-induced gene regulatory responses of glycolytic enzymes is revealed. Ablation of HIF-1α in skeletal muscle was associated with an increase in the activity of rate-limiting enzymes of the mitochondria, indicating that the citric acid cycle and increased fatty acid oxidation may be compensating for decreased flow through the glycolytic pathway in these animals. However, hypoxia-mediated HIF-1α responses are also linked to the regulation of mitochondrial dysfunction through the formation of excessive reactive oxygen species in mitochondria.

## Can You Become a Slow-Twitcher?

With the 2004 Olympics still fresh on our minds, many will ask: Who has the right stuff to go the distance? Athletes like Olympic champion Frank Shorter are clearly exceptional and represent an extreme in human skeletal muscle phenotype. Realistically, few of us can ever hope to run a marathon in world-class time. However, there may be cause for some optimism for the average mortal, since endurance exercise training in healthy humans leads to fiber-type specific increases in the abundance of PGC-1 and PPAR-α protein in skeletal muscle (Russell et al. 2003). Moreover, functional genomics support the concept that skeletal muscle remodeling to a ST phenotype, either through activated calcineurin or PPARδ, can protect against the development of dietary-induced insulin resistance (Ryder et al. 2003) and obesity (Wang et al. 2004). The results of these studies have clinical relevance since insulin-resistant elderly subjects and offspring of patients with type 2 diabetes mellitus have skeletal muscle mitochondrial dysfunction (Petersen et al. 2003; Petersen et al. 2004). Clearly, further translational studies in humans are required to test the hypothesis that increasing the proportion of ST oxidative muscle fibers will overcome the mitochondrial dysfunction and metabolic defects associated with insulin-resistant states.

## Abbreviations

FT: fast-twitch
FT_a_: aerobic FT fiber
FT_b_: anaerobic FT fiber
HIF-1α: Hypoxia Inducible Factor-1α
MAPK: mitogen-activated protein kinase
MEF2: myocyte enhancer factor 2
PGC-1: peroxisome proliferator γ coactivator 1
PPARδ: peroxisome proliferator-activated receptor δ
ST: slow-twitch
VO_2_max: maximal O_2_ uptake
